# LAYING THE REPORT FOUNDATIONS: A CONCEPTUAL MODEL OF NURSING HOME QUALITY OF CARE

**DOI:** 10.1093/geroni/igac059.227

**Published:** 2022-12-20

**Authors:** Debra Saliba, Mary Ersek, Marilyn Rantz

**Affiliations:** UCLA & GLAHS VA, LOS ANGELES, California, United States; University of Pennsylvania, Philadelphia, Pennsylvania, United States; University of Missouri Sinclair School of Nursing, Columbia, Missouri, United States

## Abstract

The committee began its work by developing a conceptual model for achieving resident-centered care. The model draws on existing evidence about the multiple dimensions of resident quality of life and the complexity of nursing home care and environments. Dr. Debra Saliba, a professor in UCLA’s Borun Center and Los Angeles VA GRECC with expertise in long-term care policy and delivery, will describe this model and how it guided the committee’s recommendations. She also will discuss how each presentation fits within the model’s domains

